# Identification of Sox6 as a regulator of pancreatic cancer development

**DOI:** 10.1111/jcmm.13470

**Published:** 2018-01-25

**Authors:** Weiliang Jiang, Qiongying Yuan, Yuanye Jiang, Li huang, Congying Chen, Guoyong Hu, Rong Wan, Xingpeng Wang, Lijuan Yang

**Affiliations:** ^1^ Department of Gastroenterology School of Medicine Shanghai General Hospital/First People's Hospital Shanghai Jiao Tong University Shanghai China; ^2^ Shanghai Key Laboratory of Pancreatic Disease School of Medicine Institute of Pancreatic Disease Shanghai Jiao Tong University Shanghai China; ^3^ Department of Gastroenterology School of Medicine Shanghai East Hospital Tongji University Shanghai China; ^4^ Department of Gastroenterology The Central Hospital of Putuo District Shanghai University of Traditional Chinese Medicine Shanghai China

**Keywords:** pancreatic cancer, Sox6, Twist1, epithelial‐mesenchymal transition

## Abstract

Pancreatic cancer (PC) is an aggressive malignancy associated with a poor prognosis and low responsiveness to chemotherapy and radiotherapy. Most patients with PC have metastatic disease at diagnosis, which partly accounts for the high mortality from this disease. Here, we explored the role of the transcription factor sex‐determining region Y‐box (Sox) 6 in the invasiveness of PC cells. We showed that Sox6 is down‐regulated in patients with PC in association with metastatic disease. Sox6 overexpression suppressed PC cell proliferation and migration *in vitro* and tumour growth and liver metastasis *in vivo*. Sox6 inhibited epithelial‐mesenchymal transition (EMT), and Akt signalling. Sox6 was shown to interact with the promoter of Twist1, a helix–loop–helix transcription factor involved in the induction of EMT, and to modulate the expression of Twist1 by recruiting histone deacetylase 1 to the promoter of the Twist1 gene. Twist1 overexpression reversed the effect of Sox6 on inhibiting EMT, confirming that the effect of Sox6 on suppressing tumour invasiveness is mediated by the modulation of Twist1 expression. These results suggest a novel mechanism underlying the aggressive behaviour of PC cells and identify potential therapeutic targets for the treatment of PC.

## Introduction

PC is the fourth leading cause of cancer‐related death worldwide 1. Although the incidence and mortality of PC have decreased in recent years, it is an aggressive malignancy with a 5‐year relative survival rate of 8% 2. For all stages combined, the 1‐year relative survival rate is only 21%. The high potential of PC cells for invasion and metastasis is the main cause of its high mortality, and approximately 80% of patients have metastatic disease at diagnosis 3, 4, 5. Therefore, a better understanding of the molecular mechanism(s) involved in the aggressive behaviour of PC is essential for the design of effective treatment strategies for this disease.

PC metastasis and treatment resistance have been associated with EMT, a process by which polarized epithelial cells acquire a mesenchymal phenotype characterized by increased migratory capacity, invasiveness and resistance to apoptosis 6, 7. EMT is characterized by the loss of the expression of the epithelial marker E‐cadherin 8. PC cells with an EMT phenotype show increased chemoresistance and cancer stem cell properties, and different growth factors and transcription factors promote EMT in PC cells 9, 10, 11. EMT is induced by the basic helix–loop–helix transcription factor Twist1, which acts together with the zinc‐finger transcription factor Snail to repress the transcription of E‐cadherin 12, 13. Twist and Snail cooperate to promote the invasive and metastatic capacity of cells in several systems.

Sex‐determining region Y‐box (SOX) 6 belongs to the SOXD family of transcription factors, which contain a highly conserved DNA‐binding high‐mobility group (HMG) domain and a group‐specific coiled‐coil domain that mediate SoxD protein dimerization and binding to pairs of DNA recognition sites 14, 15. Sox6 regulates the differentiation of tissues of mesoderm, ectoderm, and endoderm origins and modulates cell proliferation. Sox6 inhibits the growth of human colorectal cancer and hepatocellular carcinoma, and decreased expression of *SOX6* is associated with a poor prognosis in patients with hepatocellular carcinoma 16, 17. Sox6 is a negative regulator of insulin secretion from β‐cells, and its down‐regulation in the pancreatic islet cells of hyperinsulinemic mice suggests that it is involved in insulin resistance 18. However, role of Sox6 in PC development remains unclear.

In this study, we performed gain and loss of function experiments to examine the involvement of Sox6 in PC development and progression *in vitro* and *in vivo* and explored the underlying mechanisms.

## Materials and methods

### Clinical samples and cell lines

A total of 30 paired tumour tissues and 30 peritumour tissues were obtained from Shanghai General Hospital/First People's Hospital. And 20 metastatic tissues and 20 non‐metastatic PC tissues were from Shanghai General Hospital/First People's Hospital. The study was approved by the ethics committee of Shanghai General Hospital/First People's Hospital. Written informed consent was obtained from all patients. The research was carried out according to the World Medical Association Declaration of Helsinki.

The human pancreatic cancer cell lines Panc‐1 and BxPC‐3 were obtained from ATCC and cultured in Dulbecco's modified Eagle's medium (DMEM; Gibco, Grand Island, New York, USA) supplemented with 10% foetal bovine serum (FBS), 2 mM glutamine, 100 U/ml penicillin and 100 μg/ml streptomycin. All cells were incubated at 37°C in a humidified chamber supplemented with 5% CO_2_.

### Plasmid construction and lentivirus production

The human Sox6 and Twist1 coding sequences, and shSox6 (5′‐3′, CACCGCTATCACTATGGCAACTACCCGAAGGTAGTTGCCATAGTGATAGC) were cloned into the lentiviral pCDH vector. Viral production was performed in 293T cells. 293T cells were co‐transfected with the lentiviral vector pCDH and packaging plasmid. The supernatant was collected at 48 hrs post‐transfection. The viral was used to generate stably transfected cell lines.

### RNA extraction and qRT‐PCR

Total RNA was extracted using the TRIzol reagent, and mRNA was transcribed to cDNA according to the protocol supplied with the PrimerScriptRT Reagent (TaKaRa, Tokyo, Japan). The mRNA expression was analysed using SYBR Green I. Experiments were performed using an Applied Biosystems 7500 Real‐time PCR System (Foster City, CA, USA), with GAPDH as endogenous control. The PCR primers used in this study were as follows (5′‐3′): *Sox6*, TACCTCTACCTCACCACATAAGC and ACATCGGCAAGACTCCCTTTG; *E‐cadherin*, ATTTTTCCCTCGACACCCGAT and TCCCAGGCGTAGACCAAGA; *N‐cadherin*, TGCGGTACAGTGTAACTGGG and GAAACCGGGCTATCTGCTCG; *GAPDH*, CTGGGCTACACTGAGCACC and AAGTGGTCGTTGAGGGCAATG.

### Western blotting

Pancreatic cancer cells were lysed with lysis buffer (1% SDS, 50 mM Tris–HCl pH 6.8, 10 mM dithiothreitol, 10% glycerol and 0.002% bromophenol blue) supplemented with a protease inhibitor mixture (Roche, Basel, Switzerland). Equal amounts of protein were separated by SDS‐PAGE, transferred to a polyvinylidene difluoride membrane (Millipore, Bedford, MA, USA), immunoblotted with antibodies and visualized with horseradish‐peroxidase‐coupled secondary antibodies. Primary antibodies used were as follows: Sox6 and Twist1 antibodies were from Abcam (Cambridge, UK); E‐cadherin, N‐cadherin, p‐AKT and AKT antibodies were from Cell Signaling Technology (Danvers, MA, USA); β‐actin was from Sigma‐Aldrich (St. Louis, MO, USA).

### Dual‐luciferase reporter assays

A fragment of the Twist1 promoter containing the binding site for Sox6 and the mutated binding site was inserted into the pGL3.0 vector. The pGL3.0 vector containing the wild‐type (wt) or mutated Twist1 promoter was transfected into 293T cells in the presence or absence of a Sox6 overexpression vector. Thirty‐six hours after transfection, luciferase activity was detected using a dual‐luciferase reporter assay system and normalized to Renilla activity.

### CCK8 assay

Cell proliferation was assessed using a CCK‐8 assay kit according to the manufacturer's instructions (Dojindo Laboratories, Kumamoto, Japan). Briefly, cells were seeded into 96‐well plates at a density of 5000 cells per well. At the indicated times, 10 μl of the kit reagent was added to each well, incubated for 2 hrs, and cell proliferation was determined by measuring absorbance at 450 nm using a microplate reader.

### BrdU proliferation assay

BrdU incorporation assay was used to measure cell proliferation activity. Indicated cells were seeded in triplicate into 96‐well plates at density of 2000 cells per well. At 48th hour after seeding, the cells were labelled with diluted BrdU for 12 hrs. The cells were then fixed with fixative solution and incubated for 30 min. at room temperature followed by addition of anti‐BrdU antibody and incubated for 1 hr at room temperature. After washing, conjugate antibody was added and incubated for 30 min. at room temperature. After washing, the tetra‐methylbenzidine substrate was added and incubated in the dark for 15 min. Then stop solution was added, and absorbance was read at dual wavelength of 450 and 540 nm within 30 min.

### Migration assay

Transwell champers (24‐well insert; Corning, Horseheads, NY, USA) were used to analyse the ability of cell migration. The indicated cells were suspended in medium and added to the upper chambers. At the end‐point of incubation, cells on the upper membrane surface were removed. The lower membrane surface was fixed by 4% formaldehyde, stained with Hoechst and counted under a fluorescence microscope.

### Chromatin immunoprecipitation (ChIP) assay

ChIP was performed using a kit obtained from Upstate (Charlottesville, VA, USA). DNA was cross‐linked to protein using formaldehyde. Cell lysates were subjected to pulse ultrasonication to shear DNA, and immunoprecipitation was performed using antibodies against Sox6 (abcam) or HDAC1 (abcam). To clarify whether HDAC1 directly binds to Twist1 promoter, a DNA fragment which specifically interacted with HDAC1 was used as a competition inhibitor. After reversal of cross‐links, bound DNA was purified by phenol:chloroform extraction and subjected to qRT‐PCR using the following primers: Twist1 promoter, GCTCTTGGGCGAGATGAGAC and TCGGAGGAGACTGTCCTGG. Data are presented as a percentage of the input DNA.

### Tumorigenesis in nude mice

Pancreatic cancer cells (1 × 10^6^) stably expressing Sox6 by lentivirus were collected and inoculated subcutaneously into the right flank region of 4‐wk‐old male BALB/c nude mice (Institute of Zoology, Chinese Academy of Sciences, Shanghai). Tumour nodules were measured every week with a calliper. Mice were killed at the end of 3 weeks, and tumour growth and the rate of inhibition were calculated, and the tumour tissues were subjected to IHC for PCNA to detect the cell proliferation. Three independent experiments were performed for each experimental group. The animal study was approved by the ethics committee of Shanghai General Hospital/First People's Hospital.

### Animal model of liver metastasis

Liver metastasis was measured in a xenograft mouse model of PC. Pancreatic cancer cells in suspension in PBS were injected into the spleen of BALB/c nude mice. Mice were killed at 8 weeks after the injection, and the livers were excised, and metastatic lesions were photographed.

### Statistics

Statistical analysis was performed using SPSS 15.0 (SPSS Inc., Chicago, IL, USA). Values were expressed as the mean ± standard deviation (S.D.) for parametric data. Differences between groups were calculated using the Student's *t*‐test and non‐parametric test (Mann–Whitney U‐test). *P* < 0.05 was defined as statistically significant.

## Results

### Sox6 is down‐regulated in PC patients and associated with metastatic disease

**Figure 1:**
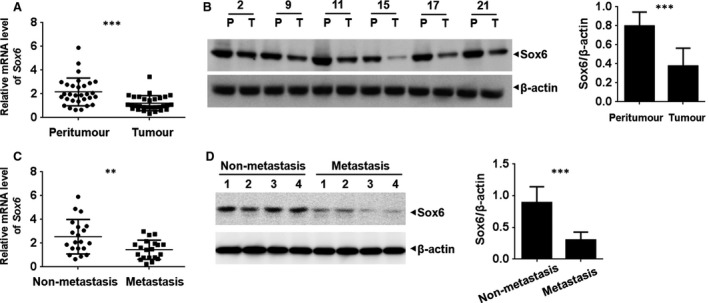
Sox6 is down‐regulated in pancreatic cancer and associated with metastasis. Sox6 mRNA and protein expressions were analysed by qPCR and Western blotting in 30 paired tumour and adjacent peritumour tissues (**A** and **B**) and 20 metastatic and non‐metastatic tumour tissues (**C** and **D**). Protein quantification was performed according to all the Western blotting graphs. P, peritumour; T, tumour. **P < 0.01; ***P < 0.001.

The mRNA and protein expressions of Sox6 were analysed in tumour and adjacent peritumour tissues from patients with PC. The results of qPCR analysis showed that Sox6 expression was lower in tumour than in peritumour tissues (Fig. 1A). Consistently, Western blot assay was carried out in 30 pairs of tissues, and protein quantification showed that protein level of Sox6 was decreased in peritumour tissues (Fig. 1B and Fig. S1A). In addition, we found that Sox6 was down‐regulated in patients with metastasis than in those with non‐metastatic disease (Fig. 1C and D; Fig. S1B).

### Sox6 suppresses PC cell proliferation and migration

**Figure 2:**
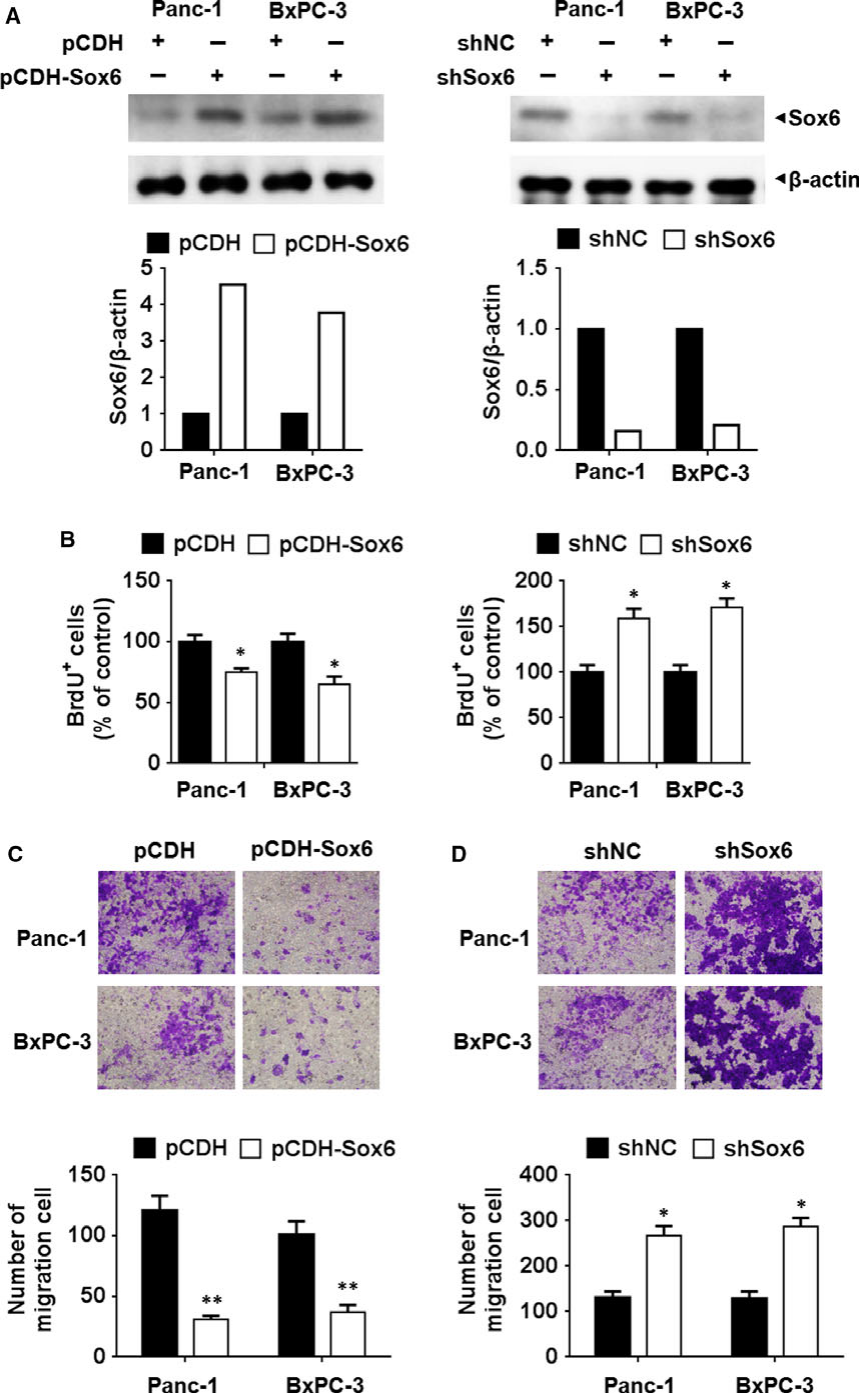
Sox6 suppressed the proliferation and migration activity of pancreatic cancer cells. The human pancreatic cancer cell lines Panc‐1 and BxPC‐3 were transfected with Sox6 overexpressing or silencing vectors. The altered expression of Sox6 was detected using Western blotting assay (**A**). Cell viability was analysed using the BrdU incorporation assay, with data normalized to control group (**B**). (**C** and **D**) Cell migration was analysed using the Transwell assay in pancreatic cells with Sox6 overexpression (**C**) or knockdown (**D**). *P < 0.05; **P < 0.01.

To examine the role of Sox6 in PC cell proliferation and migration, Sox6 was overexpressed or silenced in the PC cell lines Panc‐1 and BxPC‐3. Western blotting assay showed that Sox6 expression was successfully altered (Fig. 2A). BrdU incorporation assay showed that Sox6 overexpression inhibited cell proliferation, whereas Sox6 silencing promoted proliferation in both PC cell lines (Fig. 2B). Additionally, the results of the CCK‐8 assay showed similar results (Fig. S2A and B). Transwell assays showed that Sox6 overexpression inhibited Panc‐1 and BxPC‐3 cell migration, whereas Sox6 silencing had the opposite effect (Fig. 2C and D). Taken together, these results indicated that Sox6 plays a tumour suppressor role in PC.

### Sox6 inhibits EMT and Akt pathway activity

**Figure 3:**
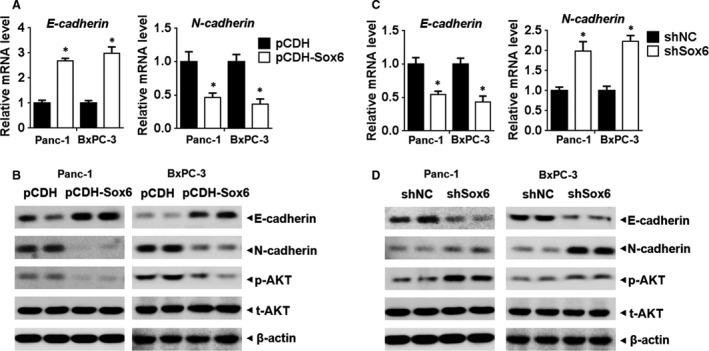
Sox6 inhibited EMT and AKT activation Panc‐1 and BxPC‐3 cells overexpressing Sox6 or with Sox6 knockdown were analysed by qPCR (**A** and **C**) and Western blotting (**B** and **D**) for the EMT markers E‐cadherin and N‐cadherin and phospho‐Akt and total Akt. *P < 0.05.

To further assess the role of Sox6 in PC, the expression of the EMT markers E‐cadherin and N‐cadherin in response to Sox6 overexpression or silencing was examined by qPCR and Western blotting. The results showed that Sox6 overexpression up‐regulated the epithelial marker E‐cadherin and down‐regulated the mesenchymal marker N‐cadherin in both PC cell lines, indicating that Sox6 overexpression inhibited EMT (Fig. 3A and B). The opposite pattern was observed in response to Sox6 silencing, as Sox6 knockdown promoted EMT (Fig. 3C and D). Western blot assessment of the levels of phospho‐Akt (p‐Akt) relative to those of total Akt showed that Sox6 overexpression down‐regulated p‐Akt (Fig. 3B), whereas Sox6 silencing up‐regulated p‐Akt (Fig. 3D), indicating that Sox6 suppressed Akt pathway activation.

### Sox6 directly interacts with the promoter of Twist1 and down‐regulates its expression

**Figure 4:**
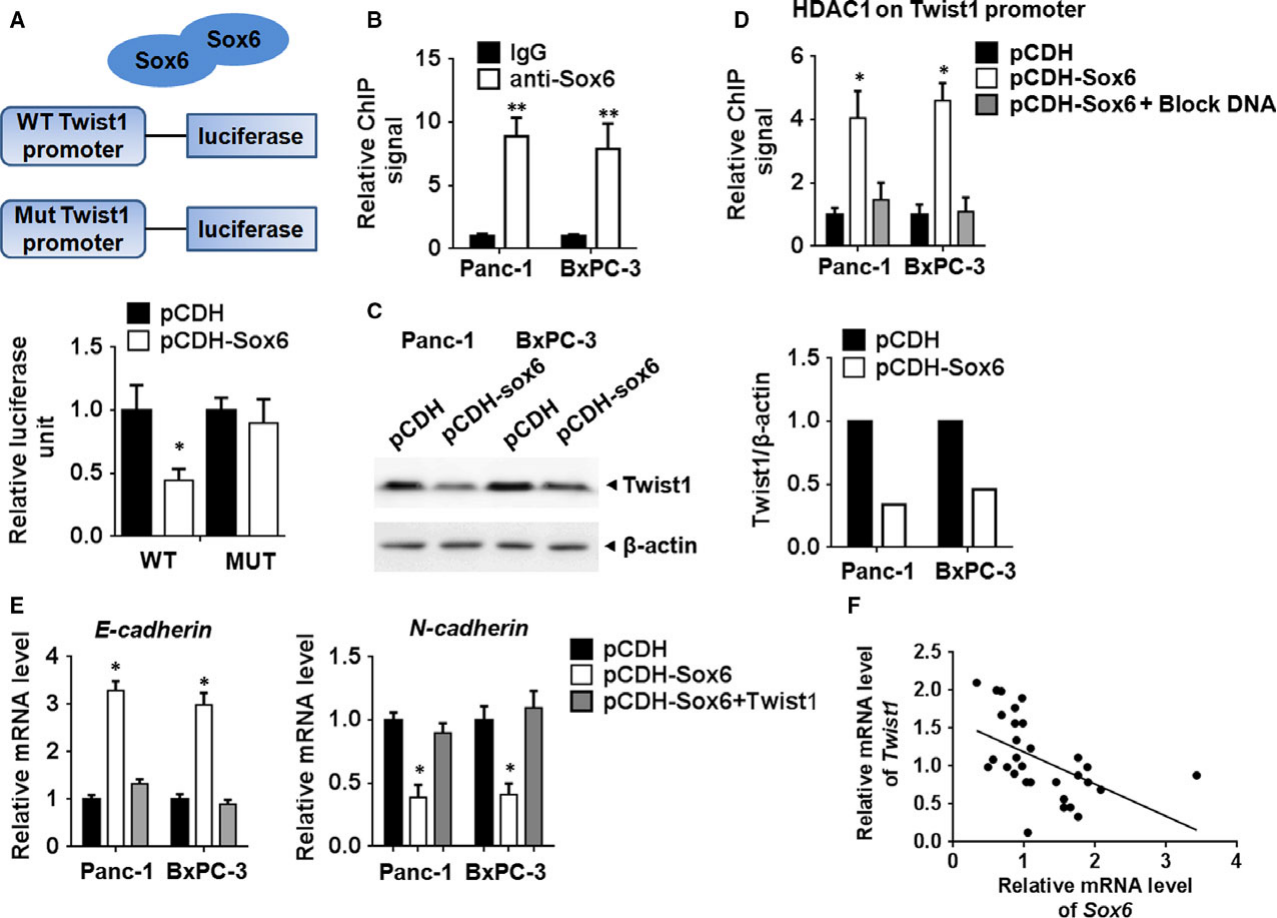
Sox6 repressed Twist1 expression by recruiting HDAC1 (**A)** The pGL3.0 vector containing wild‐type (wt) or mutated Twist1 promoter was transfected into 293T cells with or without Sox6 expression. Thirty‐six hours after transfection, luciferase activity was detected using a dual‐luciferase reporter assay system and normalized to Renilla activity. (**B**) ChIP assay showed binding of Sox6 to the Twist1 promoter. (**C**) Western blot assay of Twist1 expression in pancreatic cancer cells overexpressing Sox6. (**D**) ChIP assay showed that HDAC1 binds to the Twist1 promoter in PC cells with or without Sox6 overexpression. (**E**) mRNA expression of E‐cadherin and N‐cadherin in Panc‐1 cells overexpressing Sox6 in the presence or absence of Twist1 overexpression. (**F**) Correlation of Sox6 and Twist1 expression in PC tissues. *P < 0.05; **P < 0.01.

To investigate the mechanism underlying the effect of Sox6, we search the targets of Sox6. Several potential binding sites of Sox6 were found in Twist1 promoter (data not shown). We therefore examined it using luciferase reporter constructs bound to the wild‐type or a mutant Twist1 promoter. The results of luciferase assays showed that Sox6 overexpression significantly inhibited the activity of the wild‐type but not that of the mutant Twist1 promoter (Fig. 4A). ChIP assays showed that Sox6 binds to the Twist1 promoter in both Panc‐1 and BxPC‐3 cells (Fig. 4B). Western blot analysis showed that Sox6 overexpression down‐regulated Twist1 in Panc‐1 and BxPC‐3 cells (Fig. 4C). The regulation of Twist1 by Sox6 was further explored by ChIP assay, which showed that Sox6 overexpression promoted the binding of histone deacetylase 1 (HDAC1) to the promoter of Twist1, suggesting that Sox6 regulates Twist1 expression by recruiting HDAC1 (Fig. 4D). To clarify whether HDAC1 directly binds to Twist1 promoter, a DNA fragment which specifically interacted with HDAC1 was used. ChIP assay showed that this DNA blocked the interaction between HDAC1 and Twist1 promoter, suggesting that HDAC1 might directly bind to Twist1 promoter (Fig. 4D). qPCR experiments assessing the mRNA expression of EMT markers showed that Twist1 overexpression reversed the Sox6‐induced up‐regulation of E‐cadherin and down‐regulation of N‐cadherin, suggesting that Sox6 inhibits EMT by down‐regulating Twist1 (Fig. 4E). We then detected Sox6 and Twist1 expressions in the PC tissues, and finding out that Twist1 mRNA level was negatively related with Sox6 level (Fig. 4F). These results together suggested that Twist1 was the downstream of Sox6 and mediated Sox6 effect.

### Sox6 inhibits tumour growth and metastasis *in vivo*

**Figure 5:**
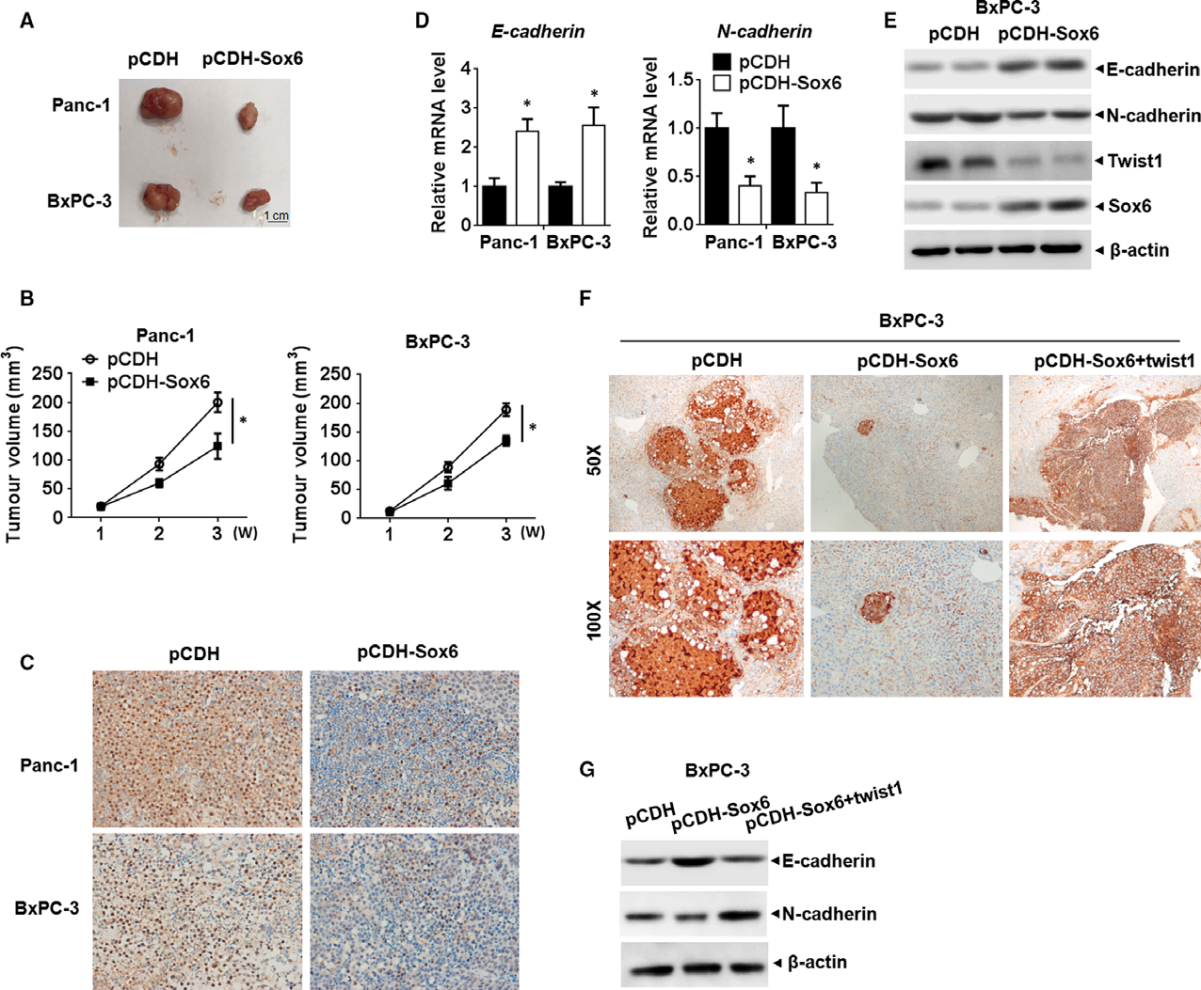
Sox6 regulated pancreatic cancer development *in vivo* (**A**). Representative images of xenograft tumours from Sox6 overexpressing or control PC cells in nude mice. (**B**) Size of the tumours was measured and calculated every week, completing growth curve of xenograft tumours in different groups. (**C**) IHC staining for PCNA in the tumour tissues developed from indicated PC cells. (**D**) qPCR analysis of *E‐cadherin* and *N‐cadherin* in the indicated group of tumours. (**E**) Western blot assay of E‐cadherin, N‐cadherin and Twist1 in the indicated group of tumours. (**F**) BxPC‐3 cells stably expression of Sox6 or Twist1 were produced and injected into the spleen of BALB/c nude mice under anaesthesia. Mice were killed 8 weeks after injection, and the livers were surgically excised and subjected to IHC staining for CEA to detect the liver lesion caused by PC metastasis. (**G**) Western blot analysis of E‐cadherin and N‐cadherin in the indicated groups of tumour. *P < 0.05.

We then investigated role of Sox6 in PC growth *in vivo*. In a mouse model of PC, mice injected with Sox6 overexpressing PC cells developed smaller tumours than those receiving control vector‐transfected cells (Fig. 5A and B). IHC assay showed that PCNA was down‐regulated in the tumour tissues developed from Sox6 overexpressing PC cells, which was a marker for cell proliferation (Fig. 5C). qPCR and Western blot assay showed that Sox6 significantly promoted E‐cadherin and inhibited N‐cadherin and Twist1 expression, suggesting Sox6 repressed EMT pathway (Fig. 5D and E). We then analysed liver metastasis by detecting expression of human carcinoembryonic antigen (CEA), which was a marker for PC. The positive area in IHC image indicated metastatic tumour tissues developed from PC cells. We found that Sox6 overexpression reduced metastasis to the liver (Fig. 5F), accompanied with impaired EMT pathway (Fig. 5G), whereas Twist1 overexpression reversed this effect (Fig. 5F and G).

## Discussion

Sox6 is a versatile transcription factor that regulates tissue differentiation and is involved in tumorigenesis in various malignancies. However, Sox6 does not contain regulatory domains, and it must therefore interact with cofactors to activate or repress gene transcription, making it a unique regulatory protein with roles throughout the life of organisms 14, 19. In the present study, we examined the role of Sox6 in PC development and progression *in vitro* and *in vivo* and elucidated a potential regulatory mechanism involving the transcription factor Twist1 and the Akt signalling pathway.

In the present study, we showed that Sox6 was down‐regulated in patients with PC and associated with metastatic disease. *In vivo* experiments showed that Sox6 overexpression inhibited tumour growth and liver metastasis from PC, confirming that Sox6 plays a tumour suppressor role in PC. Aberrant expression of certain Sox transcription factors is associated with tumorigenesis and cancer progression. Sox2 promotes cell proliferation and inhibits apoptosis in prostate cancer and is associated with the severity of disease 20. Sox4 regulates EMT in breast cancer, PC and invasive oesophageal cancer by modulating the expression of Ezh2, which encodes a histone methyltransferase 21, 22, 23. Sox5 is up‐regulated and acts as an oncogene in breast cancer, glioma and testicular seminoma 24, 25, 26. Unlike the oncogenic roles of these Sox family members, Sox6 has been reported to play a tumour suppressive role in different cancers. Sox6 is down‐regulated in hepatocellular carcinoma in correlation with poor prognosis 27. Sox6 is down‐regulated in oesophageal squamous cell carcinoma and inhibits tumour development *in vitro* and *in vivo*, acting as a tumour suppressor gene 28. The down‐regulation of Sox6 expression by microRNA‐208 in relation to increased cell proliferation and tumorigenicity in oesophageal squamous cell carcinoma supports the tumour suppressor role of Sox6 29, which is consistent with the findings of the present study.

Sox6 reduced the invasiveness of PC cells, as determined by the effects of Sox6 overexpression on inhibiting EMT *in vitro* and liver metastasis *in vivo*. The effect of Sox6 on cell migration was mediated by the down‐regulation of Twist1 through the recruitment of HDAC1 to the promoter of the Twist1 gene. Twist is a basic helix–loop–helix transcription factor that is involved in the induction of EMT together with the zinc‐finger transcription factor Snail2 30. Twist1 is overexpressed in several cancers including bladder cancer and PC, and it is associated with metastasis and cancer stem cell formation and tumorigenesis 12, 31, 32, 33. Twist1 promotes invasion and metastasis by indirectly down‐regulating E‐cadherin and inducing EMT 30, 34, 35. In breast cancer and hepatocellular carcinoma, Sox5 was shown to induce EMT by activating Twist1 expression, which is contrary to the findings of the present study with Sox6 24, 36.

In the present study, ChIP assays showed the interaction of Sox6 with the Twist1 promoter *via* recruitment of HDAC1. These results reveal a novel mechanism underlying the tumour suppressor role of Sox6 in pancreatic cancer cells. A similar mechanism was reported in pancreatic β‐cells, in which Sox6 inhibited cell proliferation by decreasing histone acetylation through the recruitment of HDAC1, resulting in the inhibition of β‐catenin‐induced cyclin D1 promoter activity 18. Future studies should further explore the role of Sox6 in the regulation of tumour cell proliferation mediated by the modulation of chromatin structure *via* histone modifications.

In conclusion, we showed that Sox6 plays a tumour suppressor role in PC, inhibiting EMT and tumour metastasis by modulating Twist1 expression through the recruitment of HDAC1 to the promoter of the Twist1 gene. These findings elucidate a potential mechanism underlying the aggressive and invasive behaviour of PC cells and identify novel therapeutic targets for the treatment of PC.

## Conflict of interest statement

The authors confirm that there are no conflicts of interest.

## Supporting information


**Figure S1** Sox6 is downregulated in pancreatic cancer and associated with metastasis. Sox6 protein expression was analyzed by western blotting in 30 paired tumor and adjacent peritumoral tissues (A) and 20 metastatic and non‐metastatic tumor tissues (B).

**Figure S2** Sox6 regulated cell proliferation by CCK8 assay. The human pancreatic cancer cell lines Panc‐1 and BxPC‐3 were transfected with Sox6 overexpressing or silencing vectors and cell viability was analyzed using the CCK8 assay at different times between 0 and 72 h. *p < 0.05.
