# Corrigendum

**DOI:** 10.1111/jcmm.17485

**Published:** 2022-08-02

In Jiang et al.,^1^ the published article contains an error in Figure 2C. The correct figure is shown below. The authors confirm all results and conclusions of this article remain unchanged.

**FIGURE 2:**
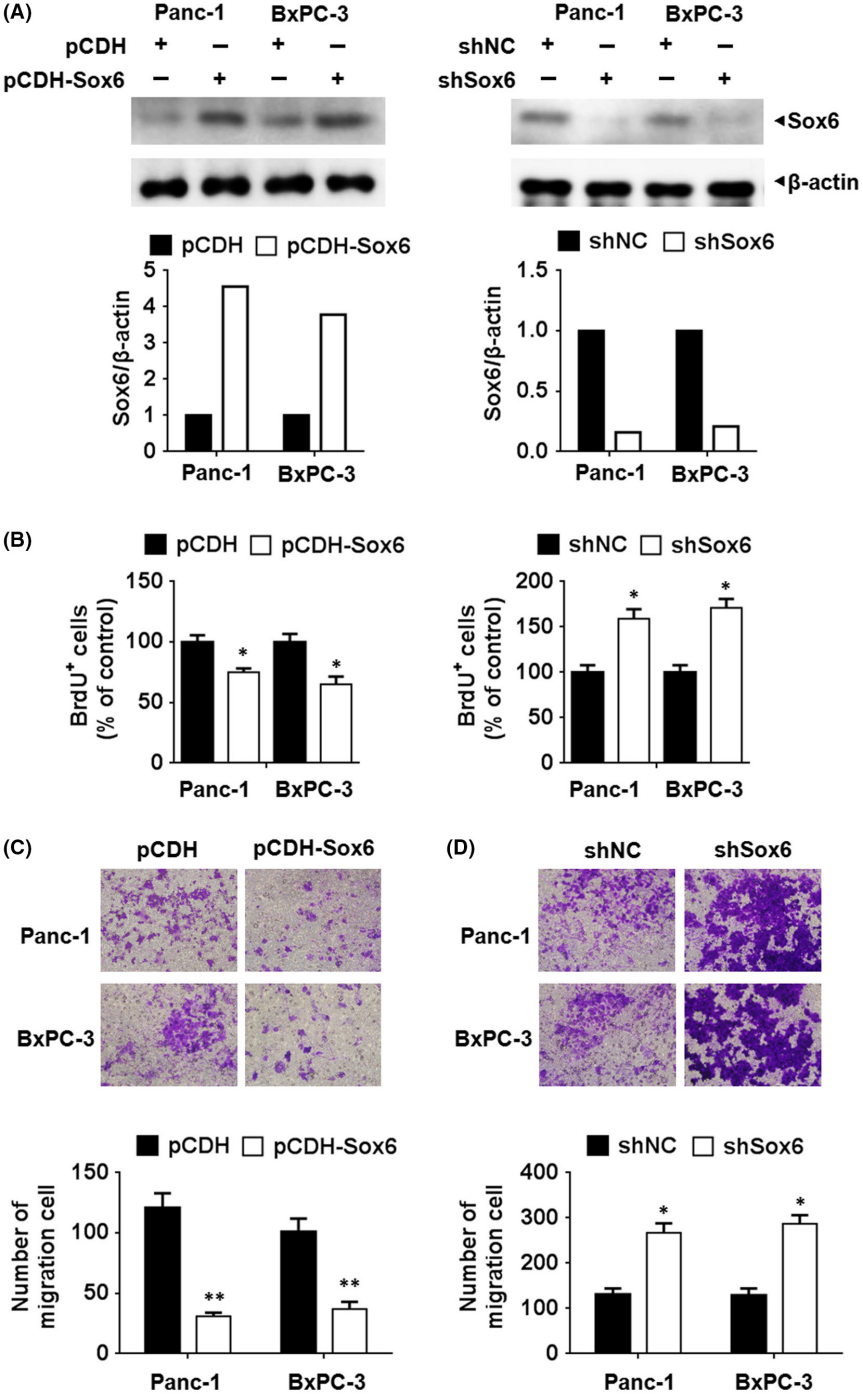
Sox6 suppressed the proliferation and migration activity of pancreatic cancer cells The human pancreatic cancer cell lines Panc‐1 and BxPC‐3 were transfected with Sox6 overexpressing or silencing vectors. The altered expression of Sox6 was detected using Western blotting assay (A). Cell viability was analysed using the BrdU incorporation assay, with data normalized to control group (B). (C and D) Cell migration was analysed using the Transwell assay in pancreatic cells with Sox6 overexpression (C) or knockdown (D). *p < 0.05; **p < 0.01

## References

[1] Jiang W , Yuan Q , Jiang Y , et al. Identification of Sox6 as a regulator of pancreatic cancer development. J Cell Mol Med. 2018;22(3):1864‐1872. 10.1111/jcmm.13470 29369542 PMC5824410

