# Supplementary figures and images for: Identification of Sox6 as a regulator of pancreatic cancer development
Source: J Cell Mol Med. 2018 Jan 25;22(3):1864–72. doi: 10.1111/jcmm.13470 (PMC5824410; doi:10.1111/jcmm.13470)

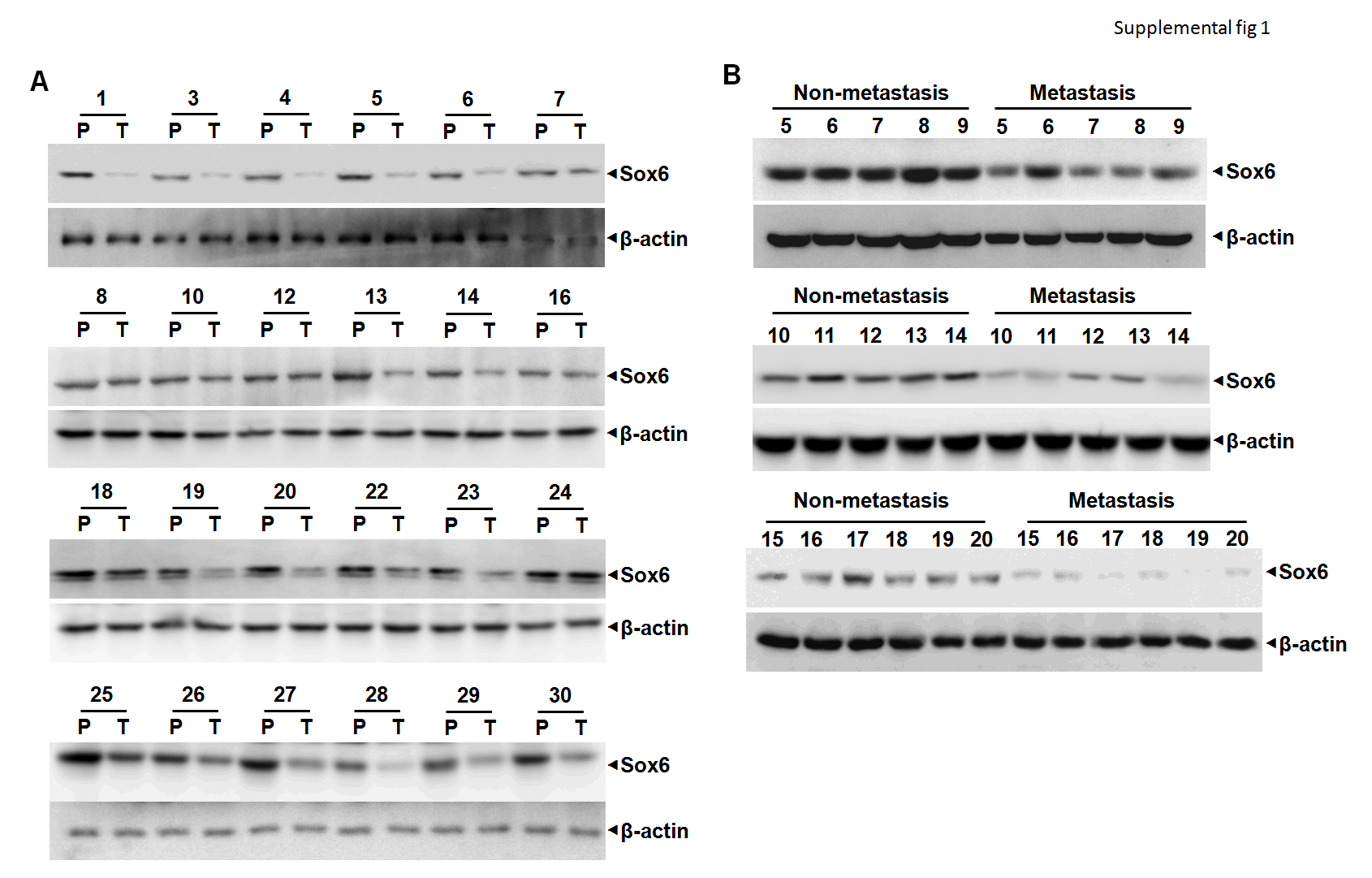

Supplement: Supplementary file 1 — Figure S1 Sox6 is downregulated in pancreatic cancer and associated with metastasis. Sox6 protein expression was analyzed by western blotting in 30 paired tumor and adjacent peritumoral tissues (A) and 20 metastatic and non‐metastatic tumor tissues (B). [file JCMM-22-1864-s001.tiff]

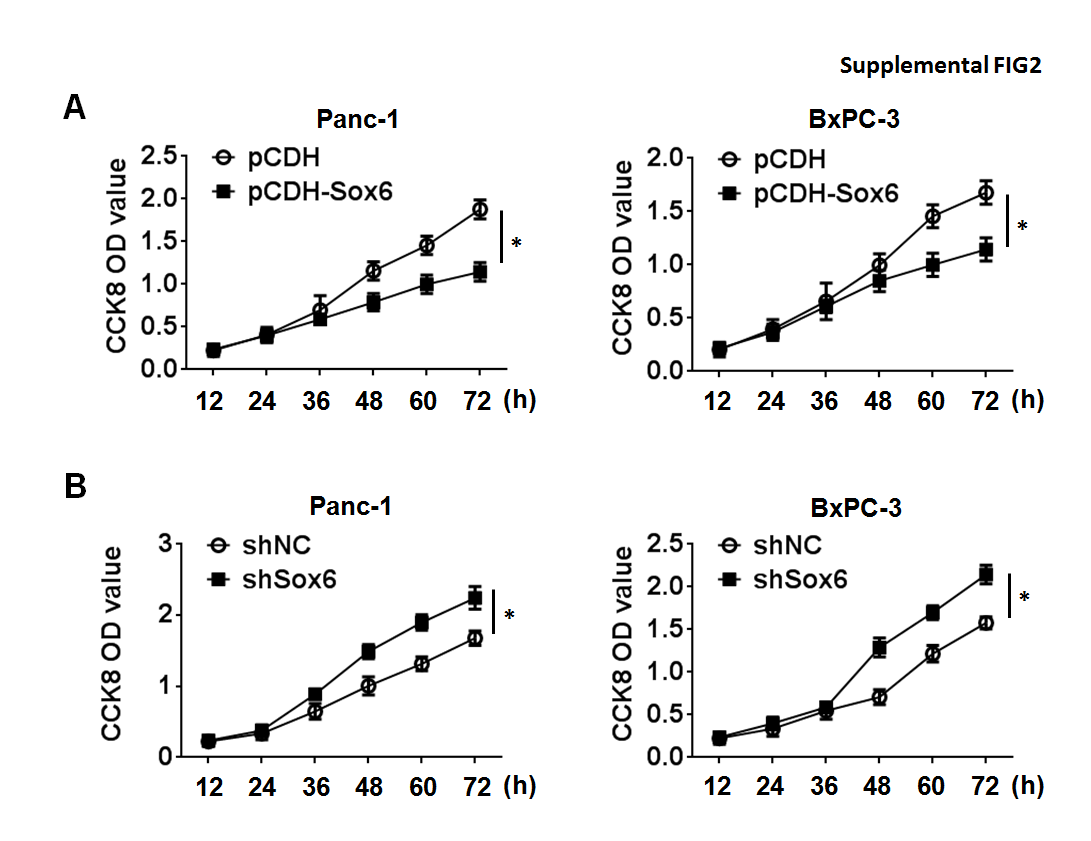

Supplement: Supplementary file 2 — Figure S2 Sox6 regulated cell proliferation by CCK8 assay. The human pancreatic cancer cell lines Panc‐1 and BxPC‐3 were transfected with Sox6 overexpressing or silencing vectors and cell viability was analyzed using the CCK8 assay at different times between 0 and 72 h. *p < 0.05. [file JCMM-22-1864-s002.tiff]
